# Cystathionine gamma-lyase of perivascular adipose tissue with reversed regulatory effect in diabetic rat artery

**DOI:** 10.1080/13102818.2014.991565

**Published:** 2014-12-18

**Authors:** Radoslava Emilova, Daniela Dimitrova, Mitko Mladenov, Teodora Daneva, Rudolf Schubert, Hristo Gagov

**Affiliations:** ^a^ Department of Animal and Human Physiology, Faculty of Biology, Sofia University St. Kliment Ohridski, Sofia, Bulgaria; ^b^ Department of Excitable Structures, Institute of Biophysics and Biomedical Engineering, Bulgarian Academy of Sciences, Sofia, Bulgaria; ^c^ Institute of Biology, Faculty of Natural Sciences and Mathematics, University of Skopje Sts. Cyril and Methodius, Skopje, Macedonia; ^d^ Department of Immunobiology of Reproduction, Institute of Biology and Immunology of Reproduction, Bulgarian Academy of Sciences, Sofia, Bulgaria; ^e^ Centre for Biomedicine and Medical Technology, Cardiovascular Physiology, Medical Faculty Mannheim, Ruprecht-Karls-University Heidelberg, Mannheim, Germany

**Keywords:** 5-hydroxytryptamine (serotonin), arterial contraction, vascular dysfunction, hydrogen sulphide, diabetes

## Abstract

The aim of this study is to reveal the regulatory role of cystathionine gamma-lyase (CSE), the main source of hydrogen sulphide (H_2_S) in perivascular adipose tissue (PVAT), of diabetic rats. Diabetes was induced in male rats by a single intraperitoneal injection of streptozotocin. Animals with glucose levels above 20 mmol/L were determined as diabetic. The rat gracilis arteries (a. gracilis) were dissected with or without PVAT. In all *in vitro* experiments endothelium-denuded preparations were used for isometric contraction measurements. Increasing concentrations of 5-hydroxytryptamine (5-HT) from 10^−10^ to 10^−5^ mol/L were applied to induce gradual increase in force of contractions of circular artery segments. The relaxing effect of CSE was inhibited by DL-propargyl glycine (PGG). The presence of PVAT decreases the contractile response to 5-HT of a. gracilis from control rats. This response is reversed in contraction studies in the same rat artery from diabetic rats. DL-PPG (1 mmol/L) induced significant increase of the force of contraction in artery preparations with PVAT from control rats in the whole range of 5-HT. In contrast, PGG had a relaxing effect in high concentrations of 5-HT (10^−6^ and 10^−5^ mol/L) in diabetic rat arteries with PVAT. It is concluded that in skeletal muscle artery from diabetic rats, a mediator related to H_2_S is released from PVAT. This paracrine mediator increases the maximal force of contraction of endothelium-denuded preparations at higher concentrations of 5-HT.

## Introduction

Adipose tissue is the largest endocrine organ, producing various adipokines and many other substances.[1] Perivascular fat, or perivascular adipose tissue (PVAT) is a thin sheet, generated during the embryonic development, which consists of adipocytes and stromal cells, including fibroblasts, leukocytes, stem cells and capillaries.[2] Almost all blood vessels are surrounded by variable amounts of PVAT associated with small arteries and arterioles.[3] PVAT together with vascular endothelium and axonal varicosities of sympathetic neurons in the adventitia, play important role in controlling the contraction of visceral [4] and skeletal muscle arteries.[5]

In this study, we focused on the effects of PVAT in diabetic rat arteries. It is well known that regulators derived from PVAT can stimulate both vasorelaxation and vasoconstriction.[6–8] Therefore, factors secreted from PVAT, like free fatty acids, adipokines, growth factors and others can directly affect the vascular function.[9] However, the rate of excretion of various adipokines may vary between PVAT at different sites in the vascular tree and between PVAT and other adipose tissue depots.[10] The role of PVAT in the regulation of blood vessels depends on metabolic state, inflammation and clinical risk factors. In health, the protective and vasorelaxant properties of the PVAT dominate, while in pathology the pathogenic influences are more evident.[11] PVAT is expanded in obesity and diabetes. This expansion does not only involve enlargement of fat cells, but also acquires macrophages of a more inflammatory phenotype.[12] Cardiovascular dysfunction is one of the complications associated with diabetes, as well as with obesity and the metabolic syndrome.[13] In mesenteric arteries, PVAT was shown to enhance constriction induced by nerve stimulation, an effect mediated by angiotensin II,[14] whereas leptin causes vasodilatation in aortic rings from Wistar-Kyoto (WKY) rats.[15] Besides vasorelaxation by PVAT of the aorta was the first vasoactive effect reported for PVAT, leading to the proposed release of an ADRF (adventitia derived relaxing factor) [7,8,16] identified as hydrogen sulphide (Н_2_S).[17] Thus, H_2_S is the third important gas transmitter in mammals, particularly in the central nervous and the circulatory systems.[18,19] H_2_S, derived from adipocytes, is synthesized in cytosol by cystathionine γ-lyase (CSE), using l-cysteine as substrate.[20] H_2_S exerts artery relaxation mainly by activation of voltage-gated potassium channels, KCNQ type, also known as Kv7 [21,22] and K_ATP_ channels.[19] KCNQ channels regulate excitability of smooth muscle cells.[23] Abnormal metabolism and functions of the CSE/H_2_S pathway have been linked to atherosclerosis and hypertension.[24] CSE knockout mice express hypertension confirming that H_2_S regulate blood pressure,[25] while substitution of H_2_S protects against the development of endothelial dysfunction.[26,27] Therefore, paracrine H_2_S signalling into the artery wall may represent a potential therapeutic target for obesity- and diabetes-associated cardiovascular dysfunction.[28] The data produced in the field can be divided into two main groups: (1) data obtained using an exogenous sources of H_2_S and (2) data obtained by stimulating endogenous H_2_S production by using the substrate L-cysteine or through targeting CSE, or by generating H_2_S from non-enzymatic reactions.[29] For example, the isolation of H_2_S-releasing and vasoactive substances from garlic may serve as a proposal of novel drugs.[30–32] Furthermore, lipophilic statins (atorvastatin) augment the vasodilatory effect of PVAT by stimulating the H_2_S production. This effect is mediated by statin-induced ubiquinone (coenzyme Q) depletion, which compromises mitochondrial H_2_S oxidation.[33] Also, under conditions of reduced H_2_S release from PVAT, its effects can be mimicked by synthetic KCNQ channel openers.[5] Аll these findings require to clarify the mechanisms of CSE-dependent regulation of PVAT in health and especially under different pathological conditions.

Therefore, the aim of our study is to reveal the regulatory role of CSE in PVAT of diabetic rat model.

## Materials and methods

### Study design

**Figure 1.**
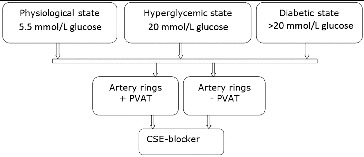
Experimental protocol.

In this study, we used two different types of preparations of rat artery gracilis – with and without PVAT (±PVAT) – under three different conditions to explore PVAT regulatory influence. The relaxing effect of CSE was inhibited by 1 mmol/L DL-propargyl glycine (PGG) (Figure 1).

### Experimental animals and induction of diabetes mellitus

Diabetes was induced in male rats by a single intraperitoneal injection of streptozotocin (STZ, 80 mg/kg body weight). STZ solution (in citrate buffer, pH 4.5) was prepared immediately prior use. Blood glucose levels for all the animals were determined by a glucometer (Gluco Chek^®^- Rapid Diagnostic PVT.Ltd., Delhi, India) at the first week after the induction and again just prior the experiments. Animals with glucose levels above 20 mmol/L were determined as diabetic.

### Measurement of isometric tension in rat *artery gracilis*

Male rats (200–300 g) were sacrificed under ether at five weeks after treatment for the induction of diabetes. The gracilis arteries (a. gracilis) were dissected and immediately transferred to cold (4 °C) physiological salt solution (PSS). The low temperature ensures the artery to be relaxed during the mounting procedure. For our research, approximately 2 mm vessel segments were selected. PVAT and connective tissue of these preparations were either removed or left intact. We used two groups of preparations from healthy male rats. They were prepared according to the same protocol as the diabetic. Then, half of them were incubated in PSS with 20 mmol/L D-glucose to evaluate the effect of hyperglycaemia. The others were kept in PSS with 5.5 mmol/L D-glucose. Before experiments with vessel rings, the normalization procedure was performed. The aim was to stretch the segment according to normal transmural pressure to ensure optimal response of the preparations. For small arteries, the target transmural pressure is 13.3 kPa. The contractile force was registered by Myodag 2.02 software (Danish Myo Technology A/S, Aarhus, Denmark).

In all *in vitro* experiments endothelium-denuded preparations were used. The endothelium was removed by gently rubbing the internal surface of the vessel segments with a rat whisker. The absence of endothelium was confirmed by the lack of relaxation to acetylcholine of 60 mmol/L KCl-contracted arteries.

All drugs and salts were from Sigma-Aldrich (St. Louis, MO, USA).

The isometric contractions were measured with Small Vessel Myograph (DMT 410M, Aarhus, Denmark). The organ bath was filled with PSS containing (in mmol/L): 119 NaCl, 4.7 KCl, 1.2 KH_2_PO_4_, 25 NaHCO_3_, 1.2 Mg_2_SO_4_, 1.6 CaCl_2_, 20 or 5.5 glucose. The bath solution was continuously oxygenated with a gas mixture of 95% O_2_ and 5% CO_2_, and kept at 37 °C; pH = 7.4. After 1 h of equilibration, the contractile force was measured under isometric conditions. The arterial contraction was expressed as a percentage of 60 mmol/L KCl-induced contraction. Increasing concentrations of serotonin from 10^−10^ to 10^−5^ mol/L were applied to induce gradual constriction of circular artery segments. All drugs were added into the bath solution (PSS).

### Statistical analysis

All data analysis were performed using statistical software SPSS 16.0. All results are given as means ± S.E.M of six separate experiments. Statistical significance was determined using Student *t*-test to assess significance between two groups or analysis of variance (ANOVA). A value of *p* < 0.05 was considered statistically significant.

## Results and discussion

Increasing concentrations of serotonin from 10^−10^ to 10^−5^ mol/L dose dependently enhance the force of contraction of all a. gracilis preparations in the three studied states.

**Figure 2.**
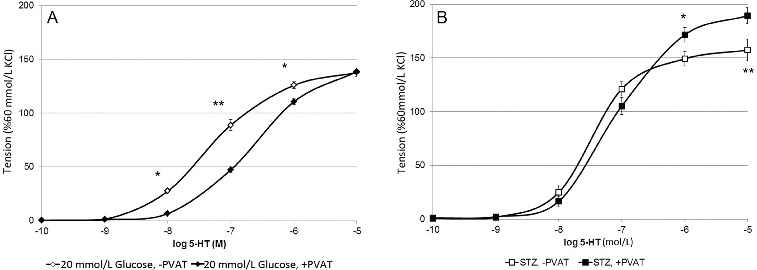
Arterial rings ±PVAT: maximal force of contraction in 20 mmol/l glucose (A) and STZ-diabetic (B) (p*< 0.05, p** < 0.01).

In the presence of a physiological glucose concentration, the 5-hydroxytryptamine (5-HT)-induced contractions of arteries with intact adipose tissue were significantly smaller at 10^−8^, 10^−7^ mol/L 5-HT (*p* < 0.001) and at 10^−6^ mol/L 5-HT (*p* < 0.01) if compared to those without PVAT (data not shown). This result consists with the data from other authors.[1,5] Similar sensitivity to 5-HT of artery rings with and without PVAT was observed when *in vitro* a. gracilis preparations were incubated in hyperglycemic conditions (Figure 2(A)). It is suggested that high glucose conditions has no influence on a. gracilis contraction. Vessel rings with or without intact PVAT of diabetic rats responded with equal contractions to 5-HT when applied in concentrations from 10^−10^ to 10^−7^ mol/L (n/s). However, at the highest studied concentrations of 5-HT, the preparations with PVAT contracted significantly stronger than those without PVAT (Figure 2(B)).These data suggest different regulatory role of PVAT in diabetic rats if compared to health controls, as well as the release of another mediator that increases the force of contraction of skeletal artery smooth muscle cells in diabetes.

When analysed the responses of blood vessels with PVAT in the three studied states, it was established that preparations from healthy rats in 5.5 mmol/L glucose and 20 mmol/L glucose were not significantly different (n/s), but diabetic rat arteries aroused significantly stronger serotonin-induced contraction (*p* < 0.01). The same results were observed when all the three groups of preparations without PVAT were compared. In control rats, the contractile effect of serotonin remained statistically unchanged in either normal or hyperglycaemic conditions (n/s). However, 5-HT contracted arterial rings of diabetic rats strongly in comparison to the controls.

This difference could be explained with decreased levels of H_2_S as a result of induction of diabetes. Thus, Whiteman et al. [34] and Jain et al. [35] reported that diabetes is associated with lower circulating levels of H_2_S. It is also known that rats with STZ-induced diabetes exhibit a decrease in their blood H_2_S concentrations without any change in the tissue expression of CSE.[36]

In a further research, we applied PGG to block H_2_S synthesis.[25] The addition of a selective inhibitor of CSE – PGG – caused a vast increase of the force of contraction of diabetic a. gracilis in the whole range of 5-HT-induced contractions in all preparations. In these from healthy rats the relaxant effect of adipose tissue was diminished. In the presence of 1 mmol/L PGG, there were no significant differences between arterial responses to 5-HT in the three tested conditions (data not shown).

**Figure 3.**
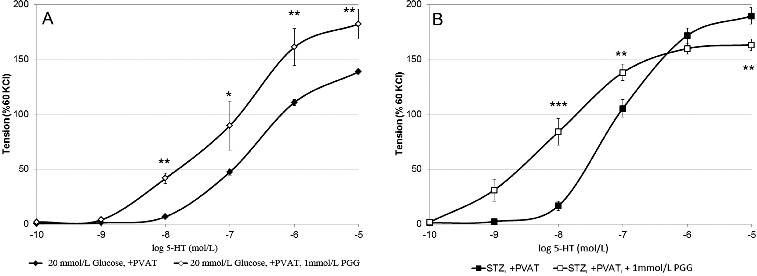
Arterial rings + PVAT ± DL-propargyl glycine: maximal force of contraction in 20 mmol/l glucose (A) and of STZ-diabetic rats (B) (*p* ^**^ < 0.01, *p* ^***^ < 0.001).

In the presence of PGG, added to block CSE, the increasing concentrations of 5-HT in the range from 10^−9^ to 10^−7^ mol/L, a. gracilis, similarly to that from healthy animals, expressed stronger contraction if compared to those without PVAT (data not shown) (Figure 3(A)). In contrast, in the presence PGG, at 10^−7^ mol/L 5-HT, the difference between PVAT-containing and PVAT-free preparations was negligible while above that value of 5-HT diabetic a. gracilis with PVAT responded with lower force of contractions (10^−6^ and 10^−5^ mol/l, Figure 3(B)). It is suggested that a second mediator causing vasoconstriction and related to H_2_S is released (probably produced only in its presence) in a. gracilis preparations with PVAT, isolated from diabetic rats. Its effect reverses the H_2_S dilatory influence and thus dominates as a common signal molecule at higher 5-HT concentrations. An alternative explanation of these surprising data is that the blockade of H_2_S production induces the generation of another relaxing mediator from PVAT under the same conditions.

## Conclusions

The presence of PVAT equally reduces the contractile response to 5-HT of a. gracilis of control animals in normal and hyperglycemic conditions. This response is converted in contraction when the same rat artery is isolated from STZ diabetic rats. The data from this study suggest that PVAT of skeletal muscle artery from diabetic rats releases a contractile mediator related to H_2_S. The nature of this signal molecule as well as the mechanism of its paracrine regulation and tissue specificity needs further elucidation. The reveal of this PVAT-smooth muscle crosstalk in the artery wall may constitute a therapeutic approach against the harmful effects of diabetes in different vascular beds.
